# An Intractable Child

**Published:** 1887-01-08

**Authors:** 


An Intractable Child.

Miss Bell is anxious to obtain another child who is perverse and subject to epileptical seizures, with a tendency to insanity, as companion to one similarly afflicted (see p. 14 of The Hospital, "F. F G."). She can offer a pretty home, with every care and attention, with school discipline.

Terms would be according to requirements.

The children would be under the supervision of a medical man, very skilled in treating such cases.

St. Mary's, Ephinstone Road,
Hastings.

